# Assessing demand for intensive care services: the role of readmission rates

**DOI:** 10.1186/cc11121

**Published:** 2012-03-20

**Authors:** RA O'Leary, B O'Brien

**Affiliations:** 1 Cork University Hospital, Cork, Ireland

## Introduction

Irish ICUs typically have bed occupancy rates approaching 100%, with 75 to 80% being the recommended level [1]. Detection of excessive demand from simple databases can thus be difficult: expedited turnover and cancellations of elective surgery often ensue, leaving occupancy rates unchanged. We hypothesised that excessive demand would produce higher readmission rates, thus illustrating the strain imposed on ICU resources during the H1N1 influenza pandemic.

## Methods

The GICU database was examined from 1 March 2010 to 1 March 2011. The H1N1 pandemic was recognised as a period of strain on the ICU and this period was estimated as 24 December 2010 to 21 January 2011. All ICU readmissions during the same hospital stay were noted. Transfers between GICU, cardiac ICU and theatre recovery were excluded as patients were still being treated by the intensive care team. Patients readmitted after transfer for extracorporeal membrane oxygenation (ECMO) were also excluded.

## Results

**Figure 1:**
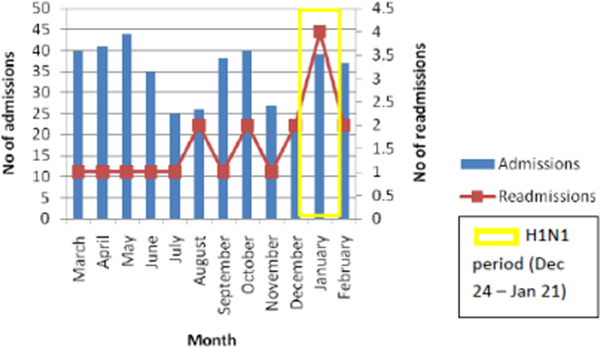
**Readmissions over time**.

The number of GICU admissions during the period was 422. There were 19 readmissions (readmission rate of 4.6%). However, this rate increased to 8.6% during the period of high activity encompassing the H1N1 pandemic (Figure 1). Hospital mortality was 36.8% in the readmission group, higher than the average, 24.6%, for the whole GICU population. This is in keeping with previous research showing up to an 11-fold increase in relative risk of mortality in patients readmitted to the ICU [2].

## Conclusion

The annual readmission rate for our unit was acceptable [3]. A clear spike was noted during the period of the H1N1 pandemic. Whilst this is a pattern we hope to address, it is a useful indicator of increased demand. Our study suggests that readmission trends in a single institution may be helpful when analysing the severity of epidemics, planning staffing needs, and comparing periods of heightened demand.
